# Rapid Chondrocyte Isolation for Tissue Engineering Applications: The Effect of Enzyme Concentration and Temporal Exposure on the Matrix Forming Capacity of Nasal Derived Chondrocytes

**DOI:** 10.1155/2017/2395138

**Published:** 2017-02-28

**Authors:** Srujana Vedicherla, Conor Timothy Buckley

**Affiliations:** ^1^Trinity Centre for Bioengineering, Trinity Biomedical Sciences Institute, Trinity College Dublin, Dublin, Ireland; ^2^Orthopaedics and Sports Medicine, School of Medicine, Trinity College Dublin, Dublin, Ireland; ^3^Mechanical and Manufacturing Engineering, School of Engineering, Trinity College Dublin, Dublin, Ireland

## Abstract

Laboratory based processing and expansion to yield adequate cell numbers had been the standard in Autologous Disc Chondrocyte Transplantation (ADCT), Allogeneic Juvenile Chondrocyte Implantation (NuQu®), and Matrix-Induced Autologous Chondrocyte Implantation (MACI). Optimizing cell isolation is a key challenge in terms of obtaining adequate cell numbers while maintaining a vibrant cell population capable of subsequent proliferation and matrix elaboration. However, typical cell yields from a cartilage digest are highly variable between donors and based on user competency. The overall objective of this study was to optimize chondrocyte isolation from cartilaginous nasal tissue through modulation of enzyme concentration exposure (750 and 3000 U/ml) and incubation time (1 and 12 h), combined with physical agitation cycles, and to assess subsequent cell viability and matrix forming capacity. Overall, increasing enzyme exposure time was found to be more detrimental than collagenase concentration for subsequent viability, proliferation, and matrix forming capacity (sGAG and collagen) of these cells resulting in nonuniform cartilaginous matrix deposition. Taken together, consolidating a 3000 U/ml collagenase digest of 1 h at a ratio of 10 ml/g of cartilage tissue with physical agitation cycles can improve efficiency of chondrocyte isolation, yielding robust, more uniform matrix formation.

## 1. Introduction

Degenerative defects in articular cartilage or cartilage-like tissues, such as disc nucleus pulposus, are a significant cause of morbidity and socioeconomic burden especially in the context of an active ageing population. While cellular repopulation in replenishing and regenerating the cartilaginous matrix has been established in the literature [1], there has been a paradigm shift in recent years, focusing on the role of primary cells or predifferentiated cells in the absence of growth factors that can maintain their phenotype in vivo [2, 3]. For example, proposed therapies for intervertebral disc (IVD) regeneration include ADCT or autologous disc cell transplantation [4] and second generation NuQU using allogeneic, juvenile chondrocyte transplantation delivered in an injectable fibrin formulation [5]. MACI or Matrix-Induced Autologous Chondrocyte Implantation is a two-step procedure involving the isolation, culture expansion, and implantation of autologous chondrocytes on a membrane or scaffold for articular cartilage repair [6]. A crucial step in these approaches is cell isolation, usually obtained through mechanical and enzymatic breakdown of a tissue biopsy and subsequent laboratory expansion in cell processing facilities.

In engineering appropriate constructs using primary cells, the need for large populations of viable chondrocytes has been a significant challenge. Cartilage is a relatively acellular tissue with only 5–10% of its volume consisting of chondrocytes [7]. In vivo, these cells reside within a pericellular matrix as chondrons [8], surrounded by dense extracellular matrix (ECM) consisting of collagens and proteoglycans. Cell yield from a cartilage digest is typically lower than 20% and is highly variable between donors and user competency [9]. Despite this, a high cell density is critical for maximising chondrogenesis [10] and remains a pertinent issue in cartilage regeneration.

In order to reconcile the low cell yield with high cell number requirements for chondrogenesis, in vitro expansion or passaging has been employed. While costly, labour intensive, and time consuming, chondrocytes can undergo a process of dedifferentiation, increasing the relative collagen type I/collagen type II production [11] which may negatively impact capacity for successful cartilage regeneration [12, 13]. This poses a significant limitation in existing regenerative therapeutic strategies using culture expanded chondrocytic cell populations.

Optimization of chondrocyte isolation is essential to enable further development of primary cell-based approaches. Limited work has been performed in this area and researchers have primarily investigated combinations of enzymatic regimes, multistep isolations, concentrations, and incubation times with different protocols [7] to improve cell yields. Previous work has investigated the role of perfusion systems in physical agitation to augment cell viability in chondrocyte isolation protocols but the role of these strategies in improving enzyme exposure is lacking [14]. When considering factors in combination, Oseni et al. investigated the necessity of a predigest phase in multistep approaches of chondrocyte isolation and found that it served no benefit in increasing the number of viable cells [7].

In manipulating the enzyme exposure in terms of concentration and incubation time, the breakdown of dense ECM which occurs gradually with time gives rise to the released chondrocytes being exposed to harsh enzymes for prolonged periods of time [15]. This reduces not only the final cell number, but also the viability and subsequent proliferative capacity of the cells [7]. While the relationship between specific digestion conditions and functional characteristics of isolated chondrocytes such as adhesion, proliferation kinetics, cell phenotype, and chondrogenic potential has been studied in rabbits, pigs, and ovine models [14], comprehensive characterization of matrix forming capacity is lacking in the literature.

Alternative chondrocyte tissue sources have also been explored, such as those from the human ear [16, 17], nose [18–21], and rib cartilage [22, 23], each demonstrating varying cell yields in line with differences in cellularity of these tissues. In particular, human nasal chondrocytes have been considered as a clinically relevant source for cartilage engineering due to the high cellularity content and regenerative potential in terms of proliferative and synthetic capacities in biochemically distinct environments from their own such as joint and disc [21–27].

The overall objective of this study was to evaluate the effect of enzyme exposure, incubation time, and additional physical agitation cycles in optimizing chondrocyte isolation from nasal cartilage biopsies using the commonly employed collagenase enzyme. Cell yield, viability, morphology, proliferation kinetics, and subsequent matrix elaboration were evaluated for the different protocol groups. In investigating the scope for cartilage regeneration using these protocols, we focused on the effect of enzyme exposure (concentration and time) on the subsequent chondrogenic potential of nasal chondrocytes.

## 2. Methods

### 2.1. Isolation of Nasal Septal Chondrocytes from Bovine Tissue and Monolayer Expansion

**Figure 1:**
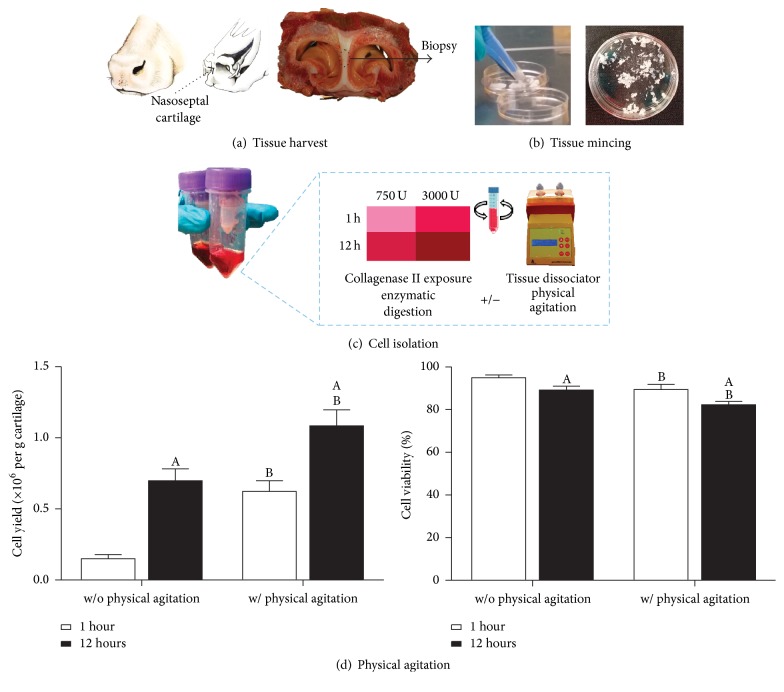
(a) Nasal cartilage was harvested from the bovine nasal septum. (b) Tissue was finely minced (~1 mm) using two scalpel blades. (c) Minced tissue was enzymatically digested using different collagenase enzyme concentrations (750 and 3000 U/ml; ratio of 10 ml per gram of cartilage tissue) and exposure times (1 h and 12 h) with (w/) or without (w/o) intermittent physical agitation using the Gentlemacs™ tissue dissociator to optimize cell yield and viability. (d) Effect of physical agitation on cell yield (×10^6^ per g of cartilage) and cell viability (%), (750 U/ml collagenase type II for 1 or 12 h incubation time, *N* = 10). ^A^Significance to 1 h of incubation. ^B^Significance compared to without (w/o) physical agitation for the same incubation period (*p* < 0.05).

Bovine nasal septa were obtained from a local abattoir within 12 h of sacrifice. Biopsies of nasal cartilage (NC) were harvested (Figure 1(a)) and washed with phosphate buffered saline (PBS) and minced (Figure 1(b)). For cell isolation, minced tissue was digested with concentrations of 750 U/ml or 3000 U/ml collagenase type II (190 U/mg, Gibco, Ireland) at a ratio of 10 ml/g of cartilage tissue for 1 h or 12 h under constant rotation at 37°C in serum-free low-glucose Dulbecco's modified eagles medium (LG-DMEM, 1 mg/mL D Glucose, 200 mM L-Glutamine;) containing antibiotic/antimycotic (100 U/ml penicillin, 100 mg/ml streptomycin) (all Gibco, Invitrogen) and amphotericin B (0.25 mg/ml, Sigma-Aldrich). The digest was subjected to physical agitation cycles at the start, after 30 min, and at the end of the incubation period using the Gentlemacs tissue dissociator (Miltenyi Biotech) (Figure 1(c)). Digested tissue/cell suspensions were passed through a 40 *µ*m cell strainer to remove tissue debris and washed three times by repeated centrifugation at 650*g* for 5 min. Cell yield and viability were determined with a hemocytometer and trypan blue exclusion. Cells were seeded at an initial density of 5 × 10^3^ cells/cm^2^ in T-175 flasks in LG-DMEM supplemented with 10% foetal bovine serum (FBS), penicillin (100 U/ml), streptomycin (100 mg/ml), and amphotericin B (0.25 mg/ml, Sigma-Aldrich). Cultures were expanded to passage one (P1) (7 d from initial isolation) in a humidified atmosphere at 37°C and 5% CO_2_.

### 2.2. Proliferation Kinetics and Cell Imaging

When subconfluent (~80%), cells were detached by treatment with 0.05% trypsin/0.53 mM ethylenediaminetetraacetic acid (EDTA) and counted using trypan blue exclusion. The number of cell doublings during the expansion phase was determined as the logarithm (base 2) of the fold increase in the number of cells during expansion. The population doubling time was defined as the culture expansion time divided by the number of doublings during the expansion phase [28].

Cells from the various isolation regimes were plated in 6-well culture plates at a seeding density of 5 × 10^3^ cells/well and cultured for 7 days. Wells were subsequently washed in PBS, fixed in 4% paraformaldehyde (PFA), and stained with hematoxylin and eosin (H&E), 2% crystal violet, or DAPI/F-Actin to assess cellular morphology and cytoskeletal filament structure.

### 2.3. Alginate Bead Encapsulation and Culture

Monolayer expanded cells were trypsinised and counted using trypan blue staining and encapsulated in 1.5% alginate (Pronova UP LVG; FMC NovaMatrix, Sandvika, Norway) at a density of 4 × 10^6^ cells/ml. The alginate/cell suspension was passed through a 12 G needle and crosslinked in 102 mM calcium chloride (CaCl_2_) to produce beads (Ø 5 mm). Beads were allowed to ionically crosslink for 20 min and subsequently transferred to 24-well plates with one bead per well and 2 ml of chemically defined medium (CDM) at 37°C with 5% CO_2_ under low oxygen (5% O_2_) conditions. CDM consisted of LG-DMEM supplemented with penicillin (100 U/ml), streptomycin (100 *µ*g/ml), 0.25 *µ*g/ml amphotericin B, 40 *µ*g/ml L-proline, 1.5 mg/ml bovine serum albumin, 4.7 *µ*g/ml linoleic acid, 1x insulin-transferrin-selenium, 50 *µ*g/ml L-ascorbic acid-2-phosphate, and 100 nM dexamethasone (all Sigma-Aldrich) with TGF-*β*3 (10 ng/ml, PeproTech, UK) supplementation. Beads were assessed at days 0 and 21 in terms of cell viability, biochemical content, and histological analysis.

### 2.4. Assessment of Cell Viability

Cell viability was assessed using a viability/cytotoxicity assay kit (LIVE/DEAD®, Invitrogen, Ireland). Briefly, constructs were cut in half and washed in phenol-free DMEM (Sigma-Aldrich, Dublin, Ireland) followed by incubation in phenol-free DMEM containing 2 *μ*M calcein AM (live, intact cell membrane) and 4 *μ*M ethidium homodimer-1 (dead, disrupted cell membrane). Sections were again washed in phenol-free DMEM, imaged with an Olympus FV-1000 Point-Scanning Confocal Microscope at 515 and 615 nm channels, and analysed using FV10-ASW 2.0 Viewer software. Quantitative analysis of cell density (per cm^2^) was determined using ImageJ software (National Institutes of Health, Bethesda, Maryland, USA) in both peripheral and core regions of the constructs and averaged for four regions.

### 2.5. Quantitative Biochemical Analysis

On removal from culture, wet weight of the samples was recorded and constructs were frozen at −85°C for further analysis. Samples were digested with 125 *μ*g/ml papain in 0.1 M sodium acetate, 5 mM L-cysteine-HCl, 0.05 M EDTA, and pH 6.0 (all from Sigma-Aldrich) at 60°C under constant rotation for 18 h followed by an additional incubation with 1 M sodium citrate under constant rotation for 1 h to disrupt the alginate calcium crosslinks. DNA content was determined using the Hoechst 33258 dye-based assay (DNA QF kit, Sigma-Aldrich, Ireland) with a calf thymus DNA standard. Proteoglycan (sulphated glycosaminoglycan, sGAG) content was quantified using the dimethylmethylene blue dye-binding assay (Blyscan, Biocolor Ltd., Northern Ireland), with a chondroitin sulphate standard. Total collagen was determined by measuring the hydroxyproline content. Samples were hydrolysed at 110°C for 18 h in 38% HCl and assayed using a chloramine-T assay [29] with a hydroxyproline:collagen ratio of 1 : 7.69 [30].

### 2.6. Histological Analysis

Beads were removed from culture, washed in PBS, and fixed in 4% PFA solution containing sodium cacodylate barium chloride (0.05 M) buffer overnight at 4°C. After removing the fixative and washing, samples were gradually dehydrated through 30–100% ethanol series with a final xylene change, before embedding in wax. Sections of 8 *μ*m were obtained with a microtome (Leica RM2125RT, Ireland) and affixed to microscope slides (Polylysine™, VWR, Ireland). Prior to staining, sections were dewaxed and rehydrated in 100% to 70% ethanol baths followed by distilled water. Cellular colonization and matrix deposition were observed using hematoxylin and eosin (H&E), sGAG deposition was evaluated using aldehyde fuchsin and 1% alcian blue 8GX in 0.1 M HCl, and collagen distribution was assessed using picrosirius red (all from Sigma-Aldrich, Ireland). Semiquantitative analysis of percentage (%) chondron in constructs was determined using ImageJ software (National Institutes of Health, Bethesda, Maryland, USA).

### 2.7. Pellet Culture Assay

To compare freshly isolated and culture expanded chondrocytes a pellet culture model was employed. Briefly, 250,000 cells of fresh and culture expanded cells isolated using the 1 h, 3000 U/ml enzyme protocol were placed in a 1.5 ml conical microtube and centrifuged at 650*g* for 5 minutes. The pellets were cultured in low-glucose chondrogenic media without additional growth factor supplementation. For histological evaluation the pellets were embedded in paraffin, cut into 5 *µ*m thick sections, and stained with 1% alcian blue 8GX (Sigma-Aldrich, Ireland) in 0.1 M HCl to assess glycosaminoglycan (GAG) content and picrosirius red to detect collagen. Subsequent biochemical analysis was carried out to quantify GAG and collagen content as outlined above.

### 2.8. Statistical Analysis

Statistical analysis was performed using GraphPad Prism (version 5) software with 3-4 samples analysed for each experimental group. One-way ANOVA was used for analysis of variance with Bonferroni's posttests to compare between groups. Results are displayed as mean ± standard deviation. Significance was accepted at a level of *p* < 0.05. The entire experiment was replicated independently with tissues from two additional donors which confirmed the findings presented in this manuscript.

## 3. Results

### 3.1. Effect of Physical Agitation in Improving Cell Yield

For a standard chondrocyte isolation protocol employing 750 U/ml collagenase type II, physical agitation was found to significantly increase cell yield (Figure 1(d)), with an almost fivefold increase after 1 h, compared with a twofold increase for 12 h exposure (*p* < 0.001). Both increased enzyme incubation time and physical agitation were found to reduce viability by approximately 6% (without (w/o) physical agitation: 1 h = 95.0 ± 1.3%, 12 h = 89.2 ±1.8%, with (w/) physical agitation: 1 h = 89.5 ± 2.4%, 12 h = 82.3 ± 1.6%).

### 3.2. Rapid Isolation and Characterization

**Figure 2:**
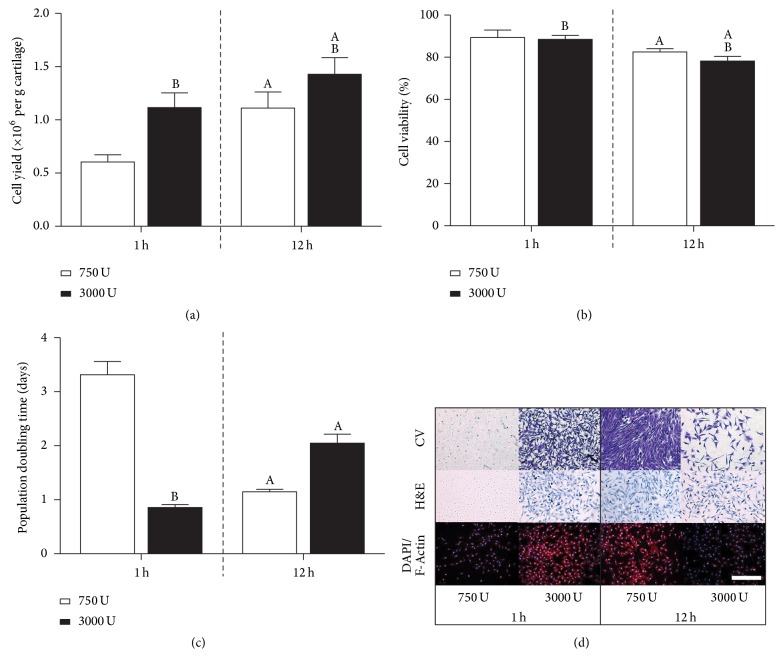
(a) Cell yield (×10^6^ per g of cartilage) for incubation times of 1 h and 12 h with 750 or 3000 U/ml of collagenase with physical agitation (*N* = 12). (b) Cell viability (%). (c) Population doubling time (days) with initial seeding density of 5 × 10^3^ cells/cm^2^. ^A^Significance to 1 h incubation for the same enzyme concentration and ^B^Significance to 750 U/ml enzyme concentration for the same incubation period (*p* < 0.05). (d) Evaluation of cell morphology with crystal violet (CV), hematoxylin and eosin (H&E), and fluorescent DAPI/F-Actin staining following 7 days of expansion (initial seeding density 5 × 10^3^ cells/cm^2^). Scale bar: 500 *µ*m.

All further experiments utilised physical agitation to determine the effect of enzyme concentration (750 and 3000 U/ml) exposure for incubation times of 1 h and 12 h. For 3000 U/ml of enzyme, the cell yield (Figure 2(a)) at 1 h was found to be similar to the 12 h digest with 750 U/ml of enzyme (*p* < 0.0001), with just over 1 million cells per gram of cartilage obtained. Minor changes in cell viability were observed for both increased incubation time and enzyme concentration exposure (Figure 2(b)). While there was a significant increase in cell yield at 3000 U/ml for a 12 h incubation time (Figure 2(a)), there was a concomitant reduction in cell viability (Figure 2(b)) (*p* < 0.0001). A 750 U/ml digest for 1 h yielded half the number of cells (*p* < 0.001) when compared with 3000 U/ml of enzyme for the same incubation time. Further, when assessing the proliferation kinetics in terms of population doubling time (Figure 2(c)), cells isolated within 1 h at 750 U/ml were found to exhibit significantly slower doubling kinetics, almost threefold slower compared with both the 1 h, 3000 U/ml and 12 h, 750 U/ml digest groups. The 12 h, 3000 U/ml group also exhibited slower proliferation kinetics (~2-fold) (*p* < 0.001). On evaluation of cell morphology with crystal violet, H&E, and fluorescence DAPI/F-Actin staining, diminished proliferative capacity was observed for 1 h, 750 U/ml and 12 h, 3000 U/ml groups (Figure 2(d)).

### 3.3. Cell Proliferation, Morphology, and Matrix Forming Capacity for Different Isolation Protocols

**Figure 3:**
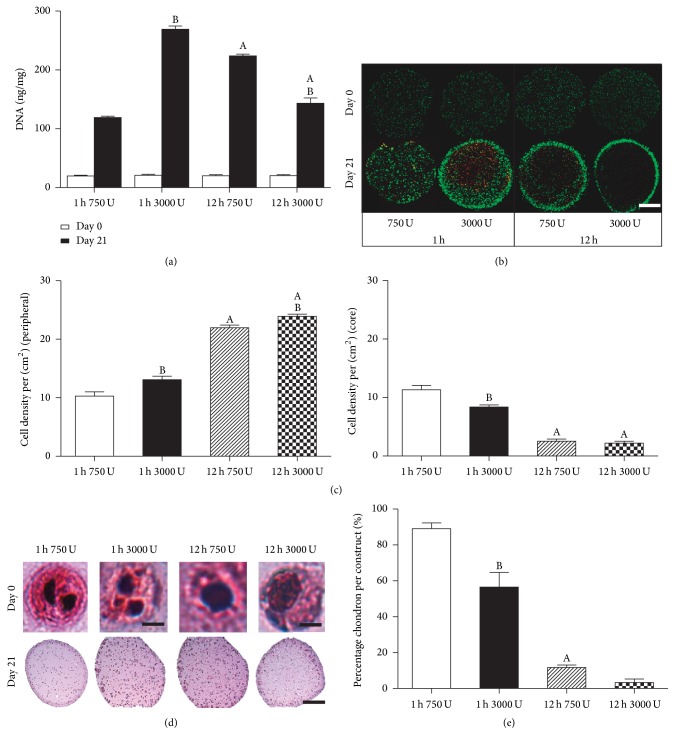
Cell proliferation and viability of nasal chondrocytes isolated using digest protocols of 1 h or 12 h with 750 or 3000 U/ml of collagenase enzyme with physical agitation and subsequent culture in alginate beads for 21 days. (a) DNA content normalized to wet weight (ng/mg) at day 0 and day 21. (b) Live/dead cell viability at day 0 and day 21. (c) Cell density (per cm^2^) for peripheral and core regions. (d) Hematoxylin and eosin (H&E) staining of cell morphology in alginate bead constructs at day 0 imaged under high magnification. Scale bar: 20 *µ*m (1 h) and 10 *µ*m (12 h) and gross alginate bead constructs at day 21. Scale bar: 1 mm. (e) Percentage chondron per construct (%) determined using ImageJ analysis. ^A^Significance to 1 h incubation for the same enzyme concentration and ^B^Significance to 750 U/ml enzyme concentration for the same incubation period (*p* < 0.05).

The trends in proliferation kinetics observed in 2D culture were also maintained in 3D alginate constructs. DNA content increased in all groups compared to day 0, with the 1 h, 3000 U/ml group exhibiting the highest DNA content (*p* < 0.001), almost twofold higher than the lower enzyme concentration (1 h, 750 U/ml) and increased temporal exposure (12 h, 3000 U/ml) groups (*p* < 0.001) (Figure 3(a)). Cell viability after 21 days was found to be dependent on enzyme incubation period with more homogenous viable cell distribution observed for cells isolated after 1 h incubation for both enzyme concentrations compared to 12 h incubation (Figure 3(b)). For cells isolated after a 12 h incubation period, a higher degree of inhomogeneity was observed in the cellular density between peripheral and core regions, with higher cell densities observed in the periphery (Figure 3(c)). Enzyme concentration exposure was also observed to have an effect on cellular distribution but to a lesser extent compared to incubation period. Cells isolated in a shorter incubation time maintained a chondron morphology compared to a single cell morphology observed with higher enzyme concentration and exposure time. Higher enzyme exposure was observed to correlate with less intense eosin staining in the pericellular matrix (PCM) indicating a reduction in PCM density (Figure 3(d)). This was also observed through semiquantitative analysis, with the highest percentage chondron being retained for the 1 h incubation and 750 U/ml enzyme concentration (Figure 3(e)).

### 3.4. Sulphated Glycosaminoglycan (sGAG) Accumulation for Different Isolation Protocols

**Figure 4:**
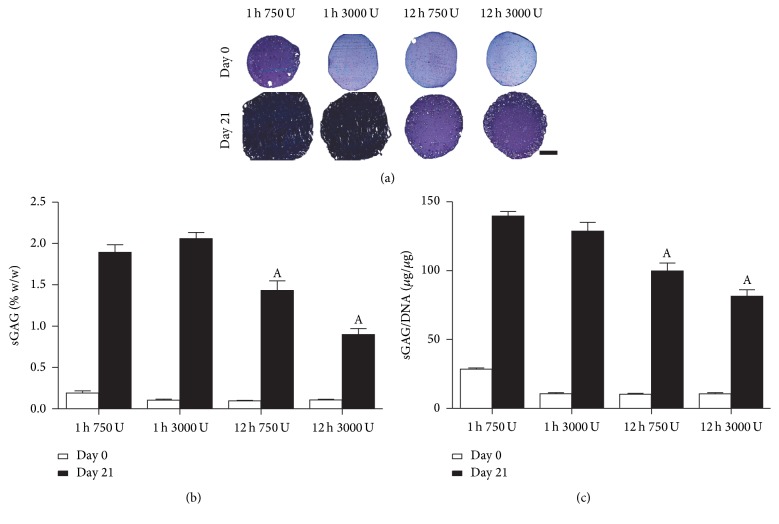
sGAG accumulation of nasal chondrocytes isolated using digest protocols of 1 h or 12 h with 750 or 3000 U/ml of collagenase enzyme with physical agitation and subsequent culture in alginate beads for 21 days. (a) Histological evaluation with aldehyde fuchsin and alcian blue to identify sGAG at day 0 and day 21; deep blue/purple staining indicates sGAG accumulation and light blue staining indicates residual alginate. Scale bar: 1 mm (b). sGAG content normalized to percentage wet weight (% w/w) and (c) sGAG normalized on a per cell basis (sGAG/DNA). ^A^Significance to 1 h incubation for the same enzyme concentration, (*p* < 0.05).

Having assessed the effect of enzyme exposure on proliferation, the matrix forming capacity was subsequently evaluated in terms of sGAG and collagen accumulation which are key constituents of cartilaginous tissues. An important difference to note in the histology at day 0 is that the 1 h 750 U/ml exposure group exhibited more intense staining for sGAG (Figure 4(a)), reflecting higher baselines sGAG (twofold) at the start of 3D culture as corroborated by the biochemical findings (Figures 4(b) and 4(c)). For both enzyme concentrations, a 1 h incubation period was found to support significantly higher sGAG accumulation compared to 12 h exposure groups (Figure 4(a)). These observations were corroborated by the biochemical analysis in terms of sGAG (% w/w) (Figure 4(b)) and sGAG/DNA (Figure 4(c)), where a significant reduction in sGAG synthesis was observed with increased incubation time.

### 3.5. Collagen Accumulation for Different Isolation Protocols

**Figure 5:**
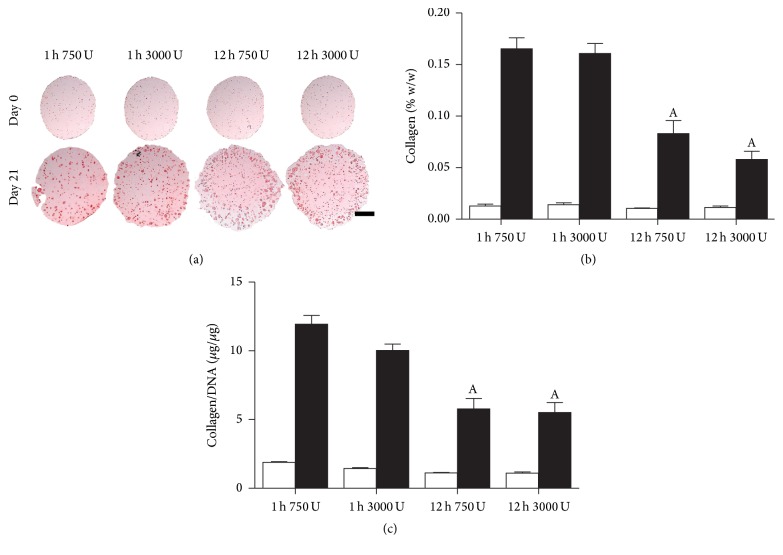
Collagen accumulation of nasal chondrocytes isolated using digest protocols of 1 h or 12 h with 750 or 3000 U/ml of collagenase enzyme with physical agitation and subsequent culture in alginate beads for 21 days. (a) Histological evaluation with picrosirius red to identify collagen at day 0 and day 21. Red staining indicates collagen deposits. Scale bar: 1 mm. (b) Collagen content normalized to percentage wet weight (% w/w). (c) Collagen normalized on a per cell basis (Collagen/DNA). ^A^Significance to 1 h incubation for the same enzyme concentration, (*p* < 0.05).

In terms of collagen accumulation, more intense histological staining was observed for shorter incubation period groups (Figure 5(a)). This was corroborated by biochemical data for both collagen (% w/w) (Figure 5(b)) and Collagen/DNA (Figure 5(c)) with significantly higher amounts of collagen, almost twofold, for shorter enzyme incubation time groups. Overall, a trend towards decreasing collagen matrix capacity is also observed with increasing enzyme exposure, with greater differences observed for increased exposure time.

### 3.6. Comparison of Freshly Isolated and Culture Expanded Chondrocytes

**Figure 6:**
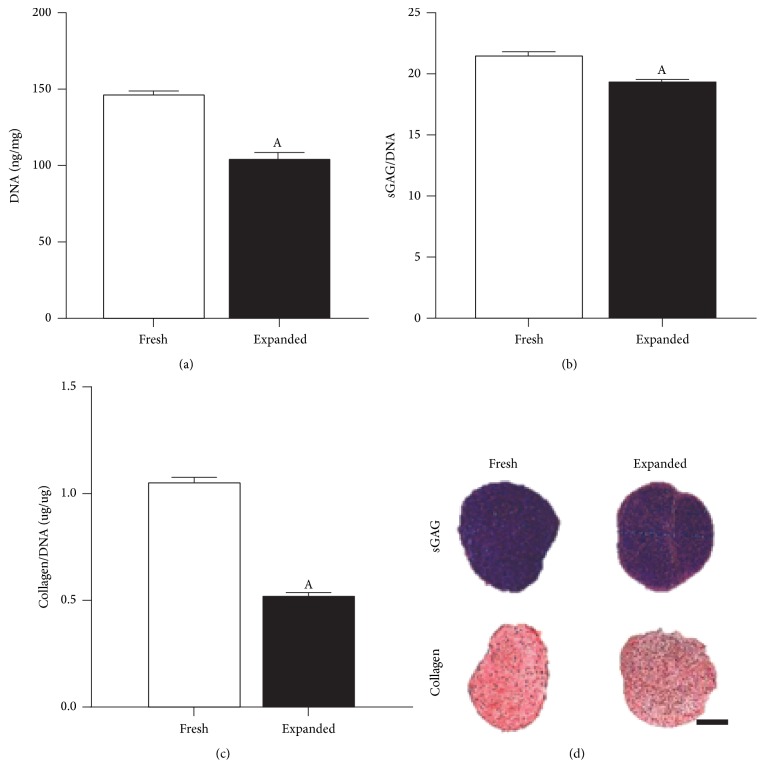
Matrix forming capacity of freshly isolated (Fresh) and culture expanded (Expanded) chondrocyte pellet cultures after 21 days. Both populations were isolated using a 1 h rapid isolation protocol with 3000 U/ml of collagenase enzyme and physical agitation. “Fresh” chondrocytes were formed into pellets immediately after isolation with “Expanded” chondrocytes being subjected to 7 days of amplification on tissue culture plastic prior to pellet culture. (a) DNA content normalized to wet weight (ng/mg) at day 21. (b) sGAG normalized on a per cell basis (sGAG/DNA). (c) Collagen normalized on a per cell basis (Collagen/DNA). ^A^Significance compared to “Fresh” group, (*p* < 0.05). (d) Histological evaluation with aldehyde fuchsin and alcian blue to identify sGAG and picrosirius red to identify collagen deposits. Scale bar: 1 mm.

Having identified that a 1 h, 3000 U/ml isolation protocol was beneficial, we next sought to compare the proliferative and matrix forming capacity of both freshly isolated and culture expanded cells in a pellet culture model system. Freshly isolated cells were found to have a higher proliferative and matrix forming capacity (Figure 6) compared to culture expanded cells, with increased DNA content (Figure 6(a)), GAG, and collagen deposition observed (Figures 6(b)–6(d)).

## 4. Discussion

In the treatment of cartilaginous defects, large populations of cells are needed for optimal chondrogenesis [10]. Optimizing cell isolation is a key challenge in terms of obtaining adequate cell numbers while maintaining a vibrant cell population capable of subsequent proliferation and matrix elaboration. In light of previous literature findings [7], the overall objective of this study was to optimize chondrocyte isolation by modulating collagenase enzyme exposure in terms of concentration and time combined with physical agitation cycles. The second objective was to evaluate the effects of enzyme exposure on subsequent cell viability and matrix forming capacity. Overall, increasing enzyme exposure time was found to be more detrimental than collagenase concentration for subsequent viability, proliferation, and matrix forming capacity (sGAG and collagen) of these cells resulting in nonuniform cartilaginous matrix deposition. Taken together, the results indicate that a 3000 U/ml collagenase digest for 1 h using physical agitation cycles can be applied as a clinically translatable protocol for isolation of chondrocytes to achieve adequate cell numbers.

The majority of collagenase enzyme concentrations utilised for cell isolation protocols are reported in terms of mg/ml or percentage weight per volume (% w/v) with typical values quoted in literature ranging from 0.08 to 0.3 (% w/v) [7]. For comparison purposes, based on the batch of collagenase used in this work (190 U/mg), 750 U/ml represents 0.4% w/v and 3000 U/ml represents 1.6% w/v. While percentage weight per volume is based on physical characteristics that are easily determined, a unit of activity is a measure of the biochemical function of the enzyme. As such, a unit of activity per gram varies for different types of collagenase or different lots of the same collagenase and can easily change over time. This inconsistency in reporting enzyme concentrations and enzyme solution to tissue mass ratios could account for much of the reported heterogeneity in isolation protocols in the literature and we advocate for consistent reporting in terms of units of enzyme in this regard.

Previous studies have investigated temperature modulation [31], human serum supplementation [32], and the use of ascorbic acid and NaCl in perfusion bioreactor systems to enhance cell isolation protocols [14]. While these approaches are highly innovative and could add significantly to advances in GMP biomanufacturing, for large scale isolation and tissue engineering approaches, short and simple protocols are desirable for clinical translation.

In considering biocompatible collagenase concentrations and minimal incubation time, physical agitation and surface area of exposure become important factors in the rapid isolation of chondrocytes. Enhancing surface area through optimal mincing and tissue breakdown can dramatically improve enzymatic action to yield similar, if not superior, cell yields [9]. Physical agitation in a cyclical fashion was shown to improve cell yield through improved tissue exposure to enzyme and increased digestion in line with the pursuits of perfusion culture systems as proposed by Centola et al. (2015) [14].

In this work, for a 750 U/ml and 1 h enzyme exposure, incomplete cell release and preservation of the chondron structure were observed resulting in lower cell yields and longer population doubling times but with superior matrix forming capacity. It is clear that balancing cell yield with viability and proliferative and subsequent matrix forming capacity specific to tissue reconstitution is key to developing optimal cell isolation protocols.

Furthermore, the improved cell viability with reduced enzyme exposure time is reflected in more uniform cell viability and matrix formation. When isolated cell populations were cultured in alginate beads, clear differences were observed between groups. Specifically, for cells subjected to longer incubation times (12 h) distinct differences in peripheral rim and core cell densities were observed, which were not as pronounced for the 1 h isolation protocol. Bos et al. (2002) demonstrated that with progressive breakdown of ECM, there is increased direct cellular exposure to enzyme which can be damaging [33]. This was observed in the changes to surrounding pericellular matrix (PCM), gradual attrition, and release of single cells with increased exposure. PCM preservation was seen to curb proliferation kinetics in the temporally less intensive enzyme regimes. We have shown here also the preservation of chondron structure in baseline constructs at day 0. PCM plays a key role in modulating the interactions of cells with the surrounding environment [34] and proliferative and synthetic responses in signaling [35, 36]. In this context, PCM plays a key role in signaling and regulation of matrix molecules [34, 37, 38]. This modulation results in lower metabolic demands [15] of these rapidly isolated cells and perhaps explains resulting homogenous matrix distribution. When considered in the context of tissue engineering or regeneration strategies, lower metabolic demands are perhaps more desirable due to compromised nutrition at the site of damage to be treated, thus making rapidly isolated cells with an intact PCM attractive for clinical translation.

Technologies such as Carti-One™ (Orteq® Ltd., United Kingdom) are currently exploiting novel intraoperative point of care (POC) cell and tissue processing. This approach allows for single staged surgery with scope for autologous tissue combined with a carrier to be delivered arthroscopically for improved repair of cartilage defects [39]. While there remain limitations, this approach highlights the role of such translatable protocols in facilitating regenerative ventures using primary cells. Further, as shown in this work, minimizing duration of enzyme exposure in a rapid isolation protocol can retain subsequent matrix forming potential.

The authors chose to work with nasal derived chondrocytes which have been proposed in the literature as an alternative primary cell source with the potential for low morbidity procurement, improved proliferation, and matrix forming capacity in cartilage regeneration [22, 23]. It is well established that culture expansion of chondrocytes results in changes in proliferative characteristics, matrix synthesis, and loss in expression of differentiation markers, termed “dedifferentiation” [40–42]. While it would have been ideal to work with fresh nasal cells for the entire study, expansion to passage 1 (P1) was necessary to obtain adequate cell numbers to demonstrate cell proliferative, live/dead characteristics, and matrix forming capacity for the various isolation protocols investigated. Having identified that a 1 h, 3000 U/ml isolation protocol was beneficial we therefore compared fresh versus culture expanded cells in a pellet culture model. In pellet culture, freshly isolated cells were found to have a higher proliferative and matrix forming capacity compared to culture expanded cells, with increased DNA content, GAG, and collagen deposition observed, further demonstrating the benefit of employing freshly isolated cells with short isolation protocols.

Cell-based medicinal products (CBMPs) follow EU legislation applicable for advanced therapy medicinal products (ATMPs) [43] with the technical requirements defined in Directive 2009/120/EC guided by the European Medicines Agency (EMA) and committee for advanced therapies (CAT) [44]. The present position of CAT considers clinical application of donor cells isolated from a different anatomical site to recipient site as “nonhomologous use” (ie., the cells or tissues are not intended to be used for the same essential function or functions) and should be classified as an ATMP requiring approval and regulation by the EMA [44, 45]. Furthermore, whereby enzymatic treatment is aimed at isolating or separating cells (which typically leads to a cell suspension with altered cell structure and functionality relative to the intact native tissue), this is considered a substantial manipulation [43] and would also require regulation as an ATMP. The development of CBMPs for clinical translation is still in its infancy and it is evident that the legislation is complex and continuously evolving with scientific advances and understanding. As the field of regenerative medicine matures and products reach commercialisation it is envisaged that the regulatory landscape may change or adapt with experience.

Future endeavours should aim at consolidating cyclical physical agitation cycles in both mincing and perfusion and modulating enzyme exposure with short incubation to yield a practical translatable protocol. Automation in a single contained unit aimed at intraoperative processing may facilitate clinically translatable strategies using chondrocytes. It should be cautioned however that further investment in these areas will be dictated by the regulatory landscape where FDA and EMA approval of point of care (POC) devices for cell isolation and intraoperative use of enzymes is necessary to apply rapid isolation of cells for use in single step approaches.

## 5. Conclusion

We recommend a 3000 U/ml collagenase digest for 1 h at a ratio of 10 ml/g of cartilage tissue with physical agitation cycles using a tissue dissociator device as a translatable protocol for intraoperative cell isolation (1−1.5 × 10^6^ cells per g of cartilage) applications. Subsequent culture of these rapidly isolated cell populations demonstrated superior proliferation kinetics, more robust matrix synthesis, and uniform matrix forming capacity. Automation of such a protocol in a single unit could facilitate single step, clinically translatable intraoperative regenerative strategies using chondrocytes for cartilage repair.
